# Education about complementary and alternative medicine in cancer self-help groups by trained peers

**DOI:** 10.1186/s12906-024-04680-2

**Published:** 2024-10-19

**Authors:** Joachim Weis, Martina Jablotschkin, Markus Horneber, Diana Steinmann, Claudia M. Witt, Stefanie Helmer, Hans Helge Bartsch

**Affiliations:** 1 Department of Self-Help Research, Medical Faculty, Comprehensive Cancer Center, University Medical Center of Freiburg, Hugstetter Str. 49, 79106 Freiburg, Germany; 2 Institute for Continuing Scientific Education, Catholic University of Applied Sciences, Freiburg, Germany; 3 Department of Internal Medicine, Division of Respiratory Medicine, Paracelsus Medical University, Klinikum Nuernberg, Germany; 4 Department of Radiotherapy, Hannover Medical School, Hannover, Germany; 5 Institute for Complementary and Integrative Medicine, University Hospital Zurich and University of Zurich, Zurich, Switzerland; 6 Institut für Gesundheits- und Pflegewissenschaft Charite Universitätsmedizin Berlin, Berlin, Germany; 7 Department of Oncological Rehabilitation (Former Medical Director), Medical Center, University Clinic Center Freiburg, Freiburg, Germany

**Keywords:** Complementary and alternative medicine, Self-help group, Oncology, Health literacy, Patient information

## Abstract

**Purpose:**

On average, about 50% of cancer patients use complementary and alternative medicine (CAM) in addition to conventional cancer treatment. Since there is a high need for information, patients often search for information about CAM and share experiences with peers, especially in self-help groups. In this study, we tested and evaluated an educational concept developed for group leaders of cancer self-help groups on how to approach the topic of CAM in their peer groups.

**Methods:**

The educational concept has been developed and piloted with health care professionals and representatives from different self-help organizations in Germany. It aims to inform and discuss the possibilities and limitations of CAM in terms of scientific evidence, to reflect user behavior, and to provide a guide on how to find and evaluate reliable and evidence-based information sources. First, self-help group leaders took part in an on-site training where they were educated on various CAM topics with the goal of being able to conduct the educational concept in their respective self-help groups. Then, the educated group leaders performed this concept in their groups. The educational concept was evaluated by both the group leaders and participants with respect to acceptance, usefulness, and satisfaction using paper-pencil questionnaires. The study was conducted in Germany based on an observational study design with a six-month follow-up. For the analyses, descriptive and univariate statistics for dependent samples were used.

**Results:**

A total of *n* = 50 interested group leaders conducted the educational program in their groups. The most common implementation barriers were organizational or structural problems, as well as the feeling of being overwhelmed with managing the program in their groups. A total of *n* = 423 participants were educated with this program by their respective group leaders. The majority felt satisfied with the educational program and improved their knowledge about CAM. At the six months follow-up (T2), significantly more participants had consulted their physicians to inform themselves about CAM and felt more confident in finding reliable information about CAM.

**Conclusions:**

In this observational study, we have managed to successfully implement an educational concept with respect to the topic of CAM in cancer self-help groups. Based on the results, the concept approach will be modified to include both a CAM- professional and group leader to train the self-help groups. In the future, the effects of the program should be tested by a randomized trial.

## Introduction

Among people with cancer, the use of complementary and alternative medicine (CAM) in addition to conventional cancer treatment is widespread and depends on clinical, psychological, and sociodemographic factors [1, 2]. In the literature, it is estimated that about 50% of cancer patients use CAM [3, 4]. There are large differences between countries, however, an overall increase in the use of CAM over the last decades has been observed. In patients with gynecological cancers, the rates are even higher up to 90% [5, 6]. Definitions of CAM vary greatly but mainly describe a variety of treatment therapies in addition to conventional medicine, e.g., natural products, cancer diets, homeopathy, phytotherapy, acupuncture, mind-body therapies, or different types of physical activity such as QiGong or Yoga. Patients with cancer or cancer survivors use CAM to improve psychological and physical well-being during and after oncological therapy or to actively contribute to their recovery [2, 7]. There is a broad range of individual motives for using CAM, e.g., to strengthen the immune system, to mitigate the side effects of conventional treatment, to influence tumor progress, or to prevent cancer recurrence [8–10]. Patients seeking CAM therapies are faced with a multitude of information presented online, in print media, or by health care providers [11]. Moreover, the use of different CAM methods is often recommended by family members or friends and mostly based on insufficient information [12]. Therefore, discriminating between sound and questionable or harmful CAM therapies and making an informed decision is often difficult for patients.

Based on increasing interest in CAM and the attempt to integrate CAM into conventional cancer care, national and international evidence-based guidelines for the use of CAM in oncology have been published [13–16]. Seeking information about CAM on the Internet may often be frustrating for patients and their relatives due to the lack of transparency and scientific evidence [17]. Some studies have shown that cancer patients wish to discuss the use of CAM with their medical doctors and want their support in the decision-making process [18–23].

As a result of increasing research activities in this field, the scientific evidence for CAM methods has improved but is still limited. Nevertheless, the integration of CAM therapies into cancer care is still in its early beginning. Oncologists often reject CAM [24, 25] not only because of the lack of scientific evidence but also because of certain risks, for example, the interaction of micronutrients with oncological treatment including hormone therapy or modern therapies such as checkpoint inhibitors [26–28]. Some studies have shown that patients want to be informed about CAM options by their doctors, however, they usually get this information from other sources [29–31]. One such source of information is the exchange and sharing of CAM experiences by cancer patients with their peers, e.g., in cancer self-help groups (SHG) or peer-to-peer conversation. This information, however, may not be reliable or based on scientific evidence [32–34]. In Germany, cancer self-help groups are an important source of information and psychosocial support for patients and cancer survivors [35].

In this context, the overall aim of our project was to design an educational concept that supports cancer patients in self-help groups to improve the peer-to-peer conversation about CAM and provide them with reliable information about CAM. This study was part of the Collaborative Research Network of Complementary Medicine in Oncology (KOKON) which focused on CAM information and supportive programs aimed at improving consulting skills for healthcare professionals and information about CAM for cancer patients [36, 37].

The aim of the study was to implement and evaluate this educational concept. The following research questions were addressed:

1. Was the educational concept suitable to provide the SHG leaders with the necessary support enabling them to discuss CAM in their groups?
2. Did the group members benefit from this concept by improving their information level of information and knowledge about CAM?

## Methods

In a previous concept development study [38] we developed and pre-tested both the educational concept and a structured manual for group leaders (GLs) in collaboration with four major German self-help organizations (SHOs): women with cancer (breast, gynecological) (FSH), prostate cancer (BPS), colon cancer and stoma carriers (ILCO) and hematological malignancies (DLH) [39]. Based on this preparatory work, we now implemented and evaluated the educational concept in this study. The study procedure consisted of two parts: First, we trained group leaders (GLs) to educate the group participants (PTs) about CAM. Secondly, the educated group leaders (GLs) used the manual provided to relay information about CAM and discuss CAM therapies in their peer groups. This paper focuses on the results of the PT evaluation of the educational sessions led by the trained GLs in the SHGs.

We evaluated the program on the level of GLs and PTs by means of self-developed questionnaires with various statements to be rated using Likert scales from 1 to 6 (does not apply at – fully applies), 1 to 6 (1 = extremely satisfied to 6 = not satisfied), or school grading from 1 to 6 (1 = very good to 6 = inadequate). For the subjective rating of knowledge on the subject of CAM, we used a 3-level Likert scale (1 = very good, 2 = moderate, 3 = low). On the GL level, we asked for their confidence in the CAM topics with regard to the four modules: the organizational feasibility, the applicability of the educational concept for their SHG, implementation difficulties, and the quality of the informational material provided. The evaluation of the educational concept presented by the GL in their groups included a participant survey immediately after the educational session (T1) and a follow-up after six months (T2). At T1, the PTs evaluated the comprehensibility of the topics, the possibility of asking questions and sharing experiences, the moderation by the GL, the informational material that was provided, and the group atmosphere. At T2, PTs evaluated the benefit of the education with respect to improvement in their knowledge about CAM and changes in their attitude towards CAM. In addition, we assessed how frequently PTs used the recommended information sources and how helpful this was for them. In addition, sociodemographic data and the affiliation to the various self-help organizations (SHOs) were assessed.

### The CAM educational concept

The manual included four modules: (1) Overview of CAM methods and potential interaction with conventional cancer treatment, (2) Expectations of cancer patients towards CAM, (3) Criteria for the appraisal of CAM methods, and (4) Reliable sources for web-based CAM information. Four qualified websites for cancer information were recommended: The German Cancer Information Service (Krebsinformationsdienst) [40], KOKONinfo (Website of the KOKON Network) [41], Onkopedia (Website of the German Scientific Association of Hematology and Oncology) [42], and the website “Integrative Medicine” (Memorial Sloan Kettering Cancer Center) [43]. Each module included a 15-minute lecture followed by a discussion period of approximately 30 min to discuss participants‘ experiences as well as questions. In addition, the GLs were provided with a written guide on how to handle the manual, a set of PowerPoint slides for all four modules, and a paper brochure for each group member. When presenting the educational concept in their groups, the GL could make use of the slides depending on the technical equipment in their meeting locations and their own preferences. In addition, they were provided with the program manual and brochures for the participants. GLs were also allowed to vary the arrangement of the four modules according to the time schedule or the interest of the group.

The aim of the GL training was to enable the GLs to present the four modules mentioned above and facilitate a structured discussion about various aspects of CAM with the group members. Participants should be motivated to improve their knowledge and to learn where to find reliable information about CAM. It is important to mention that the GLs were not trained to become CAM experts or to give any CAM recommendations to individual group members. For this purpose, the manual included a list of certified websites for CAM.

### Recruitment of participants (group leaders and group participants)

The implementation of the educational program was realized in cooperation with the SHOs mentioned above with the exception of the DLH which declared to have no further interest in deepening the topic of CAM in their groups through this study. The education of a GL took an average of three hours and was carried out by two members of the research group with sound CAM knowledge (a health educator, a psychologist, and three oncologists). The training was offered on a voluntary basis. For practical reasons, it took place as a part of the regional SHO meetings of the SHOs.

### Statistical analysis

The data were mainly analyzed descriptively using IBM SPSS© version 25.0. Furthermore, differences between groups and correlations were calculated. For all cross-sectional analyses of categorical variables, a Chi-square test or a McNemar test was applied. To determine the correlation between age and the evaluation items, a Spearman correlation was calculated, and a Mann-Whitney-U-test was applied to detect gender differences in the evaluation of the training. Differences between the participants based on the three self-help organizations or on the educational level were analyzed by one-way ANOVA and Games-Howell or Tukey post-hoc tests or Welch’s ANOVA when variance homogeneity was not met. Wilcoxon tests for dependent samples were performed to evaluate the changes at the six-month follow-up (T2).

## Results

### Description of the sample

Out of *N* = 577 GLs, who were educated during 19 SHO meetings with a group size between 13 and 80 GLs, *n* = 229 GLs (39.7%) were interested in implementing the CAM education in their SHGs. Finally, *n* = 50 GLs (21.9% of the interested GL) conducted the CAM educational program in their groups between November 2017 and June 2019 (see Table 1). The GLs who did not carry out the concept reported a lack of interest in CAM by the group members as well as various organizational problems as reasons why the educational program was not implemented in their groups. Table 1 shows that the average age of the *n* = 50 GLs was 64.7 years (SD 10.0); 58% were female. Most GLs, *n* = 30 (60.0%), belonged to the FSH, *n* = 14 (28.0%) to the BPS, and *n* = 6 (12.0%) to the ILCO. The majority of the GLs graduated from high school (65.9%). Using a documentation sheet, the GLs stated that out of a total of 915 group members, *n* = 434 PTs (47.4%) took part in the educational sessions. We received the questionnaires from a total of *n* = 423 PTs (97.5%). Most of the PTs were members of the FSH (61.7%), followed by the BPS (25.1%), and the ILCO (11.3%). The average age of the PTs was 65.9 years (SD 10.9). The majority were female (63.6%) and had a higher educational level (see Table 1).

**Table 1:** Sociodemographic characteristics of the group leaders (GL) and participants (PT) of the course in the self-help groups

GL (*N* = 50)				PT T1 (*N* = 423)		PT T2 (*N* = 211)	
Age (years)		Mean (SD): 64.7 (10.0) *n* = 41 ^d^		Mean (*SD*): 65.9 (10.9) *n* = 388 ^d^		Mean (SD): 66 (11.4) *n* = 206 ^d^	
		**n**	**%**	**n**	**%**	**n**	**%**
*Gender*		*n* = 49 ^d^		*n* = 414 ^d^		*n* = 206 ^d^	
Female		29	59.2	269	65.0	124	58.8
Male		20	40.8	145	35.0	82	41.2
*Self-help organization*		*n* = 50 ^d^		*n* = 415 ^d^		*n* = 209 ^d^	
FSH^a^ (breast and gynec)		30	60.0	261	61.7	112	53.1
BPS^b^ (prostate cancer)		14	28.0	106	25.1	61	28.9
ILCO^c^ (colon cancer)		6	12.0	48	11.3	36	17.1
*Education (graduation)*		*n* = 41 ^d^		*n* = 412 ^d^		*n* = 207 ^d^	
High level (> 10 years)		27	65.9	155	37.6	87	41.2
Secondary school (*≤* 10 years)		6	14.6	144	35.0	62	29.4
Secondary school (5–9 years)		3	7.3	85	20.6	43	20.4
Others		5	12.2	28	6.8	15	7.1

^a^ FSH (German Federal Association Women’s Self-Help Cancer), ^b^ BPS (German Federal Association for Prostate Cancer Self-Help), ^c^ ILCO (Federal Self-Help Association for patients with stoma and colorectal cancer and their relatives; ^d^ Valid cases

#### Evaluation of the CAM educational program by the trained group leaders at T1

The GLs needed on average 90 min (SD 31; range 43 to 180) for the CAM educational program in their groups. The majority (62.0%) only used the paper brochure, and 38.0% used the PowerPoint presentation as well. In addition, the time available to discuss each module was evaluated to be just right. As Table 2 shows, the GLs were able to carry out the educational program as provided, appreciated the slides for presenting the topics, and felt confident in moderating the discussion about CAM in their groups. The GLs also rated the active involvement of the participants, the group atmosphere, the interest of the participants in CAM, and the implementation of the educational program overall as being good (Table 2). In addition, they were asked to what extent they had already dealt with the subject of CAM before the course.

**Table 2:** Evaluation of the education program by the GL at T1 (*N* = 50)

How would you evaluate the education ^a^	*N*	MW (SD)
I was able to impart the learning material by the provided slides	49	2,06 (0,92)
I felt confident in moderating the group discussion	50	2,14 (0,93)
The manual and recommendations for moderating were helpful for me	50	1,86 (0,76)
The provided material for the program was helpful for me	50	1,84 (0,68)
**How would you evaluate the following aspects of the implementation with school grading** ^b^	**N**	**Mean (SD)**
… the active involvement of the participants?	50	2,04 (0,86)
… the group atmosphere?	50	1,80 (0,70)
… the interest in CAM of the participants?	50	2,18 (0,92)
… the implementation of the education overall?	48	2,18 (0,74)

^a^ 1 = fully applies to 6 = does not apply at all; ^b^ school grading from 1 to 6 (1 is very good and 6 is inadequate)

#### Evaluation of the CAM educational program by group participants at T1

In the evaluation of the educational program presented by the GLs, the PTs appreciated the contents and the understandability of the modules (see Table 3). They felt that the modules provided useful information for their personal situation and that the informational material was helpful. Overall, they would recommend the CAM educational program to other participants of the self-help group. Comparing the four modules, there were no substantial differences in how the participants evaluated the various aspects. In addition, the educational program stimulated a process of reflection for PTs on how to deal with CAM, provided information about where to find reliable CAM information, and how to assess the credibility of CAM providers. Participants also learned about possible pharmacological interactions between specific CAM therapies and the standard treatment. Overall, they were very satisfied with the educational program, and the majority (90.8%) of the PTs would recommend the training to other peers. In evaluating the educational program, no significant differences were found between men and women, the type of SHO, or the level of education.

**Table 3:** Evaluation of the training in the SHGs by the PT across all four modules (T1) (*N* = 423)

Items ^a^	*N*	Mean (SD)
The contents of the modules were good ^a^	359–397	2.15 (0.79–0.83)
The contents were easy to understand ^a^	367–401	2.13 (0.74–0.79)
The modules provided useful information for my personal situation ^a^	367–397	2.51 (0.96–1.11)
The information material was helpful ^a^	390–402	2.26 (0.85–0.86)
I would recommend the CAM education to other participants of the self-help group ^a^	366–403	2.08 (0.84–0.91)
I am satisfied with the CAM education ^b^	367–396	2.25 (0.75–0.82)
**Overall assessment of the education**		
**The training …**	***n***	**Mean (SD)**
… helped me to reflect my own attitudes towards CAM ^a^	420	2.27 (0.96)
… showed me new aspects of CAM ^a^	419	2.36 (0.99)
… informed me where to find reliable information on CAM ^a^	415	1.97 (0.78)
… helped me how to check the credibility of providers of CAM^a^	418	2.00 (0.76)
… taught me that pharmacological interactions of CAM with standard therapy may exist ^a^	414	2.08 (0.86)
… did not bring me anything new ^a^	410	3.52 (1.44)
… left me with some unanswered questions ^a^	399	3.68 (1.32)

^*a*^ 1 = fully applies to 6 = does not apply at all. ^b^ 1 = extremely satisfied to 6 = not satisfied

With regard to the overall assessment of the training, the PTs rated the various criteria of the educational program, namely the benefit for the individual situation, the presentation of contents by the group leader, the possibility to share their experiences and to make their own comments, the group atmosphere, and the informational material, as being “good” based on a school grading scale (1 = “very good” to 6 = “insufficient”).

### Results of T1 and the six-month follow-up (T2)

Six months after the educational program, *n* = 211 PTs (49.9%) responded to the follow-up questionnaire. Sociodemographic data and affiliation to the type of SHO were comparable to T1 (see Table 1).

At T1, more than half (52.1%) of the PTs stated that they had used complementary medicine products or applications and wanted to expand their knowledge of CAM. Before the educational program, they informed themselves about CAM by asking other members of the SHG (83.9%), through books, magazines, or flyers (51.1%), or by searching the internet (40.0%). In addition, 38.5% of the PTs discussed CAM with a physician other than their oncologist, 33.8% with other cancer patients, 27.4% with friends or family members, 22.9% with oncologists, and 12.5% with alternative (CAM) practitioners (multiple answers possible). Compared to T1, the ranking of the CAM information sources utilized remained the same, but significantly more PTs (49.8% vs. 38.7%) stated that they had consulted their physicians and oncologists more often to inform themselves about CAM (McNemar test: x2 = 7.11, *p* = .008, *n* = 211). At follow-up, 37% reported that they had accessed the internet for CAM information, with 45% using the websites recommended in the program three to five times in the period following participation in the program. The majority of PTs used a qualified website for cancer information: 87.4% accessed the Krebsinformationsdienst [40], 55.8% the KOKONinfo [41], 43.2% Onkopedia [42], and 20.0% the website from Memorial Sloan Kettering Cancer Center [43] (multiple answers possible). User satisfaction and the level of support provided by the recommended websites were perceived as good. Those PTs who did not use websites named reasons such as “no need”, “no internet access/lack of PC skills”, “health/family problems”, “no time”, and “the use of other information sources”.

As Table 4 shows, the confidence to distinguish reliable from unreliable information is slightly lower at T2 than at T1. The intensity of dealing with the topic of CAM remained unchanged.

**Table 4:** Evaluation of the program comparing after psychoeducation (T1) and six months later (T2) (*n* = 211)

How do you rate the following statements?	*N* Valid cases	T1 Mean (SD)	T2 Mean (SD)	df	*p*	d
I am dealing with the subject of CAM ^a^	207	2.99 (1.60)	2.80 (1.24)	206	n.s.	
I like to expand my knowledge in handling CAM ^a^	204	2.16 (1.01)	2.55 (1.01)	203	≤ 0.001	− 0.11
I feel confident in distinguishing reliable from unreliable CAM information ^a^	202	1.92 (0.79)	2.88 (1.29)	199	≤ 0.001	0.70
I have still questions about CAM ^a^	183	3.74 (1.31)	3.76 (1.27)	189	n.s.	
My knowledge on the subject of CAM is …. ^b^	208	2.23 (0.63)	2.08 (0.60)	-	≤ 0.01	− 0.24

^*a*^ = mean values based on 6-level Likert scale from 1 = fully applies to 6 = does not apply at all; ^b^ = mean values based on 3-level Likert scale: 1 = very good, 2 = moderate, 3 = low

The mean of the item “I like to expand my knowledge in dealing with CAM” significantly decreased from 2.16 (sd: 1.01) at T1 to 2.55 (sd: 1.01) at T2 (*p* ≤ .001) (see Table 4).

The confidence searching for information on complementary medicine significantly decreased from T1 (mean: 1.92 sd: 0.79) to T2 (mean 2.88 sd: 1.29) (*p* ≤ .001) which illustrates that even after the educational program, this remains a challenge for the PTs. At T2, the mean of the item “Still questions about how to deal with CAM” was still on a moderate level (mean 3.76 sd: 0.27) and showed no changes compared with T1 (mean: 3.74 sd: 1.31).

At T1, the individual CAM knowledge was rated as very good by 11.3%, moderate by 53.9%, and low by 34.8% (mean: 2.23 sd: 0.63). In comparison to T1 directly after the program, the knowledge of CAM was rated significantly higher at T2 (mean: 2.08 sd: 0.60) and the wish to expand one’s knowledge about CAM was significantly lower (Table 4).

## Discussion

In this study, an educational concept for SHG leaders on how to discuss CAM issues within their SHGs was successfully implemented in three SHOs. We trained GLs on how to instruct the group members about CAM and to moderate the resulting discussions in their groups. To our knowledge, this train-the-trainer concept for CAM with representatives of SHGs is unique. Compared to the concept development study [38], some modifications were made to meet the needs of the various SHGs, e.g., the option of technical support using a PowerPoint presentation. The flexible applicability of the concept is reflected by the wide range of implementation times for the course and the personal preference for use of the PowerPoint slides. An important element of the concept is the sharing of the group members´ experiences with CAM and an open discussion about the pros and cons of CAM. Through the support of the training concept, this open exchange, in combination with concrete assistance on how to identify questionable or harmful treatments and drugs and optimize the search for online-based information, can help people to reflect on their own attitudes towards CAM and their use of it and, if necessary, to adjust these. The evaluation results indicate that after the training, a process of reflection on the use of CAM was initiated. There was a significant increase in the number of PTs who sought information about CAM from their physician in the period after the course. In a pilot study carried out in Australia, the effect of evidence-based CAM education, including written information and a video, was investigated in a sample of *N* = 20 cancer patients. An impact of this education was observed showing a decrease in intent to use CAMs in cancer care of 14%. Thus, the decision-making process took into consideration the information and recommendations provided through the education [21]. The few studies that have been conducted to date on the effect of reliable CAM information materials do not yet al.low generalized conclusions to be drawn. Nevertheless, these initial results seem to indicate that they can be an effective support in the use of CAM. Although an increase in knowledge is detected and there is no urgent need to discuss the CAM topic any longer in the sample investigated in this study, uncertainties in the search for reliable information were stated. The reasons could be that websites were recommended for the information search, however, the use of such websites could be overwhelming or unattractive for a portion of the PTs, e.g., due to limited digital literacy. Also, the selection of recommended websites, the duration of the follow-up period, or even the complex topic of CAM itself, which requires having to understand and deal with an unclear evidence base, could have encouraged a certain restraint in the evaluation of the training concept. We also think that the education requires a thematic deepening of the complex topic of complementary medicine to achieve stronger effects. In addition, this could motivate participants to ask individual questions regarding their own therapy and lifestyle. According to the feedback from numerous GLs, the schedule for planning the educational program in some of the SHOs was too short as most SHOs plan the education program for their meetings more than one year in advance.

### Limitations

As we performed an innovative approach in close collaboration with cancer patient self-help organizations, we were faced with some difficulties. Although we had *n* = 229 GLs who were interested after the educational program, only *n* = 50 GLs (21.9%) implemented the training in their own SHG. The main reasons were various organizational or motivational problems. The training was only implemented in a limited number of SHOs. In addition, the final sample of GLs and PTs was not balanced in terms of the SHOs and included a majority of female GLs. Therefore, the results cannot be generalized to other cancer patient self-help organizations. There is also some evidence that those of the GLs who did not follow through to implementing the program in their SHG felt over-challenged presenting the CAM modules and moderating the discussion within the group.

## Conclusion

The evaluation shows that the educational concept with GLs can help to address the complex issues of CAM within the self-help groups. We were able to achieve the two aims of this study, but we still suggest some modifications to the concept. To improve our concept, we plan to include a professional expert in the educational program to support the GLs. In addition, we would consider offering the courses independently from the existing group meetings and including E-learning modules to achieve more flexibility and stronger effects of the education for the participants. For the future, the modified program should be tested with a randomized study design.

## Data Availability

The data that support the findings of this study are not openly available due to reasons of sensitivity and are available from the corresponding author upon reasonable request.
